# Offshore Finfish Aquaculture in the United States: An Examination of Federal Laws That Could be Used to Address Environmental and Occupational Public Health Risks

**DOI:** 10.3390/ijerph111111964

**Published:** 2014-11-19

**Authors:** Jillian P. Fry, David C. Love, Arunima Shukla, Ryan M. Lee

**Affiliations:** 1 Johns Hopkins Center for a Livable Future, Johns Hopkins Bloomberg School of Public Health, 615 N. Wolfe Street, W7010, Baltimore, MD 21205, USA; E-Mails: dlove8@jhu.edu (D.C.L.); arunima89@gmail.com (A.S.); rlee89@jhu.edu (R.M.L.); 2 Department of Environmental Health Sciences, Johns Hopkins Bloomberg School of Public Health, 615 N. Wolfe Street, Baltimore, MD 21205, USA; 3 Department of Health, Behavior and Society, Johns Hopkins Bloomberg School of Public Health, 624 N. Broadway, Baltimore, MD 21205, USA

**Keywords:** Exclusive Economic Zone, federal regulations, fish farming, food production, food safety, occupational health, ocean policy, offshore aquaculture, public health, seafood

## Abstract

Half of the world’s edible seafood comes from aquaculture, and the United States (US) government is working to develop an offshore finfish aquaculture industry in federal waters. To date, US aquaculture has largely been regulated at the state level, and creating an offshore aquaculture industry will require the development of a new regulatory structure. Some aquaculture practices involve hazardous working conditions and the use of veterinary drugs, agrochemicals, and questionable farming methods, which could raise environmental and occupational public health concerns if these methods are employed in the offshore finfish industry in the US. This policy analysis aims to inform public health professionals and other stakeholders in the policy debate regarding how offshore finfish aquaculture should be regulated in the US to protect human health; previous policy analyses on this topic have focused on environmental impacts. We identified 20 federal laws related to offshore finfish aquaculture, including 11 that are relevant to preventing, controlling, or monitoring potential public health risks. Given the novelty of the industry in the US, myriad relevant laws, and jurisdictional issues in an offshore setting, federal agencies need to work collaboratively and transparently to ensure that a comprehensive and functional regulatory structure is established that addresses the potential public health risks associated with this type of food production.

## 1. Introduction

Aquaculture, or farmed seafood, has experienced rapid growth over the past few decades and now accounts for about half of seafood consumed worldwide [1]. The United States (US) government aims to expand the domestic aquaculture industry in light of a large seafood trade deficit, fast growth in the aquaculture industries of other countries, fully exploited or declining wild fisheries in most parts of the world, the potential for economic growth, and national dietary guideline recommendations to increase seafood consumption [2,3].

**Figure 1:**
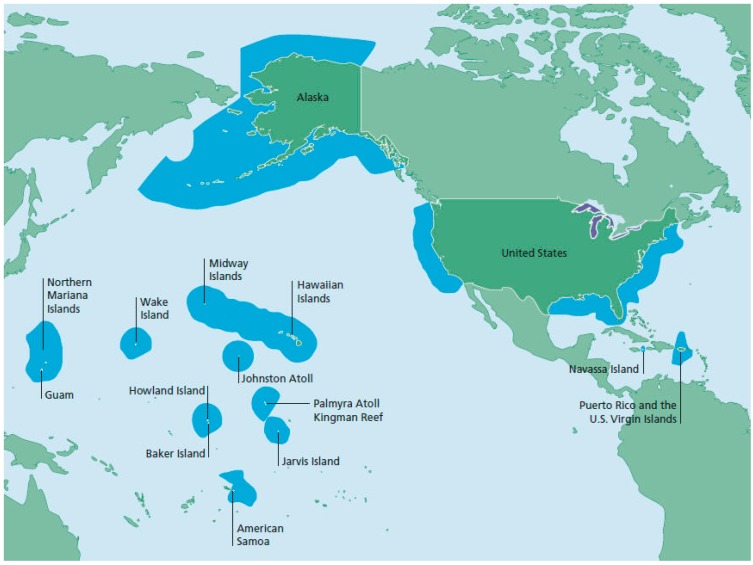
The US Exclusive Economic Zone is the largest in the world. Source: NOAA Fisheries Service.

The US government is interested in developing offshore aquaculture, especially in the federally controlled Exclusive Economic Zone (EEZ). The US EEZ, the largest in the world, starts at the territorial sea (12 nautical miles offshore) and extends up to 200 nautical miles offshore (Figure 1) [4]. The focus of this paper is offshore finfish aquaculture; the term finfish refers to fish and not shellfish or crustaceans.

In offshore finfish aquaculture, fish are raised in net-pens and floating or submerged cages. As of 2007, 24 countries have had near- or offshore aquaculture operations or demonstration projects raising finfish and shellfish [5]. In the US, near- or offshore aquaculture operations and/or pilot projects have been located off the coast of California, Hawaii, New Hampshire, Washington, Maine, Puerto Rico, and the Gulf of Mexico [5,6], but large-scale commercial offshore production has not developed.

Less than one percent of global aquaculture production takes place in the US [1]. In 2013, there were about 2500 US farms with edible aquaculture production, totaling $1.15 billion in sales, and roughly half of this production was finfish (primarily catfish, but also trout, tilapia, yellow perch, and hybrid striped bass) [7]. There is a small, near-shore Atlantic salmon aquaculture industry with a half-dozen farms [7], which takes place in waters regulated by the states of Maine and Washington and federal agencies. The responsibility of regulating an offshore aquaculture industry in the EEZ would fall mostly to federal agencies [8]. Congress failed to pass legislation introduced in 2007 and 2011 aimed at establishing a regulatory framework for offshore aquaculture [9,10]. Instead of a federal law designed to regulate offshore aquaculture, a patchwork of laws exist that may have relevance to the issue [8]. The result is a complicated regulatory situation, which has been cited as a barrier to commercialization [11]. The Gulf of Mexico Fisheries Management Council, a regional body responsible for managing fishery resources, has recently pursued a path to permit offshore aquaculture in the region’s EEZ [12], but they cannot move forward until federal agencies publish regulations relevant to their legal authority. The National Oceanic and Atmospheric Administration (NOAA) has published a report on minimizing environmental impacts of offshore aquaculture through best management practices [13], and the agency is expected to publish regulations indicating the requirements for offshore aquaculture in 2014 [14]. The policies under development should be designed to minimize negative public health and environmental consequences of offshore aquaculture production practices [15].

### Public Health Concerns Relevant to Offshore Aquaculture

Offshore finfish aquaculture operations have the potential to affect aquatic animals and environments through transmission of diseases, fish escapes, uneaten fish feed, waste effluents, and veterinary drug and agrochemical use [16,17,18,19,20]. Many of these issues impact public health in direct and indirect ways. If veterinary drugs and agrochemicals are used to control pests and treat or prevent disease, they may become incorporated into fish tissue or promote the development and spread of antimicrobial resistant bacteria [21,22,23]. A small proportion of farmed seafood is inspected at national borders, and some samples are found to be above tolerance values for veterinary drug residues, metals, and microorganisms [24]. In addition, feeds made with fishmeal and fish oil can contain persistent organic pollutants (POPs) and heavy metals, as has been reported in European farms [25]. These contaminants are present in the ocean, in part due to anthropogenic pollution, and are biomagnified as they move up the aquatic food chain [25,26]. Many of these risks can also impact food safety of nearby wild fish caught for human consumption because offshore finfish aquaculture operations generally do not have the ability to prevent chemicals and veterinary drugs (if used) and uneaten feed and fish waste from leaving the farm environment and flowing into adjacent waters [15,27].

Aquaculture workers in the US suffer elevated rates of non-fatal injuries, similar to agriculture workers [21], and an offshore aquaculture industry could present greater hazards to workers in the US due to the offshore setting. The most significant occupational risks associated with inland, near-, and offshore finfish aquaculture include exposure to drugs, agrochemicals, pathogens, and extreme temperatures; falls from boats and cages; breathing dust from feed; musculoskeletal injuries; needle-stick injuries; and diving risks including decompression illness and drowning [21,28].

Offshore aquaculture regulations related to siting, operation size, stocking density and other factors can help mitigate public health concerns, for example by reducing pollution caused by the use of drugs, chemicals, uneaten feed, and fish waste [13,29]. There are many relevant laws that could provide the basis for these and other regulatory controls, and our analysis aims to increase understanding of how current federal laws could be used to address public health risks associated with production and consumption of finfish farmed offshore and consumption of wild seafood caught nearby.

## 2. Methods

We conducted a literature and document review from February to August 2013 to identify US federal laws relevant to offshore finfish aquaculture. We started by reviewing the peer-reviewed scientific literature for public health risks associated with near- and offshore finfish aquaculture using the search engines/databases Google Scholar, PubMed, and ScienceDirect. A list of potential public health risks from offshore finfish aquaculture was developed and then we searched for laws relevant to those risks. To identify US federal laws, we read and analyzed relevant legal reviews, government documents, journal articles, and reports by nongovernmental organizations (NGOs) about US aquaculture. We identified these documents using the search engines Google and Google Scholar. We created a list of laws that were identified in Excel (Microsoft), and then we researched each law using documents and reports from government and non-government sources to determine the relevance to offshore finfish aquaculture in the US and applicability to issues impacting public health. This approach enabled us to identify laws that have not been included in reviews focused solely on environmental risks.

We categorized the laws based on their potential to address aquaculture practices that may threaten public health (low *vs.* high). Purely environmental impacts of offshore aquaculture that are less relevant to public health (*i.e*., entanglement of endangered species in nets/cages) are not the focus of this paper. In addition, laws related to seafood harvesting and processing were only reviewed if they were germane to aquaculture (*i.e*., monitoring of drug and chemical residues).

## 3. Results 

We identified 20 laws relevant to offshore finfish aquaculture in the US EEZ. Nine of the 20 laws had a low potential to address aquaculture practices that may pose risks to public health, because they are aimed at addressing issues such as protection of animals designated as endangered species, preventing illegal trade of wild animals, establishment of marine sanctuaries, and requiring permits for structures that may interfere with navigation. These laws, summarized in Table A1, are potentially important for managing offshore aquaculture in the US, but they will not be useful for addressing potential public health issues. The remaining 11 laws are more relevant in regard to offshore aquaculture production practices that may pose risks to public health. Each law is described in Table 1 and the text below, organized by six lead agencies and one law with multi-agency jurisdiction.

**Table 1:** Federal laws relevant to potential public health issues associated with offshore finfish aquaculture.

Federal Law	Lead Federal Agency	Offshore Aquaculture Issue(s) Potentially Addressed by Law	Relevant Public Health Issue(s)
Magnuson-Stevens Fishery Conservation and Management Act	National Oceanic and Atmospheric Administration (Department of Commerce)	Various issues could be addressed through setting limitations on offshore aquaculture permits	Antibiotic useFood safetyOccupational health and safety
Clean Water Act	Environmental Protection Agency	Limiting and monitoring pollutants released into the ocean through issuing National Pollutant Discharge Elimination System (NPDES) Permits	Antibiotic useFood safety
Ocean Dumping Act	Environmental Protection Agency	Control or limits on the dumping of chemicals, veterinary drugs, feed, and/or waste into the ocean	Antibiotic useFood safety
Federal Insecticide, Fungicide and Rodenticide Act	Environmental Protection Agency	Restrictions on the sale and labeling of pesticides	Food safetyOccupational health and safety
Toxic Substance Control Act	Environmental Protection Agency	Restrictions and/or requirements for reporting, record keeping, and testing for new and existing chemicals and mixtures	Food safetyOccupational health and safety
Federal Food, Drug, and Cosmetic Act and relevant amendments	Food and Drug Administration (Department of Health and Human Services)	Regulation and approval of animal drugs and feed additives, control of drug and pesticide residues in food products, reporting of veterinary drug use in animal production	Antibiotic useFood safetyOccupational health and safety
Occupational Safety and Health (OSH) Act	Occupational Safety and Health Administration (Department of Labor)	Set exposure limits, require use of personal protection equipment, reporting of incidents, and other occupational issuesM Note: OSH Act jurisdiction effectively ends at the territorial sea because regulations giving jurisdiction over activities in the Outer Continental Shelf preempt the OSH Act.	Occupational health and safety
US Coast Guard (Code of Federal Regulations; Title 46, Chapter 1)	US Coast Guard (Department of Homeland Security)	Safety of individuals working aboard certain vessels	Occupational health and safety
Virus-Serum-Toxin Act	US Department of Agriculture	Monitoring the quality and safety of veterinary biologics	Food safetyOccupational health and safety
Animal Health Protection Act	US Department of Agriculture	Monitor diseases among edible farmed fish due to food safety issues and the potential for farmed fish to pass diseases onto wild seafood speciesNote: Effective disease control can reduce the use of harmful drugs and chemicals in aquaculture	Food safetyOccupational health and safety
National Environmental Policy Act	Varies	Analysis of proposed actions that may have an impact on the quality of the environment	Antibiotic useFood safetyOccupational health and safety

### 3.1. National Oceanic and Atmospheric Administration (Department of Commerce)

#### Magnuson-Stevens Fishery Conservation and Management Act 

The Magnuson-Stevens Act (MSA) gives NOAA’s National Marine Fisheries Service the authority to regulate fishing in federal waters. Under the MSA, eight Regional Fishery Management Councils were established and given responsibility for developing Fishery Management Plans (FMPs), in collaboration with NOAA, aimed at preventing overfishing, maintaining optimal catch levels, and meeting other goals [30]. NOAA’s interpretation of the MSA includes aquaculture as a form of “fishing” [12].

FMPs may be used by Regional Fishery Management Councils to set limitations on offshore aquaculture permits. There are some important requirements included in the Gulf of Mexico FMP about fish species, NOAA oversight, and adaptive management that may address some environmental public health concerns, even if they are addressed indirectly [12]. On the other hand, since the MSA was designed to regulate the capture of wild fish, the law itself is not designed to ensure the safety of feed, control the use of drugs and chemicals, or monitor and limit fish escapes and pollution from offshore aquaculture sites.

### 3.2. Environmental Protection Agency

#### 3.2.1. Clean Water Act 

The Environmental Protection Agency (EPA), under jurisdiction granted by the Clean Water Act (CWA), oversees the National Pollutant Discharge Elimination System (NPDES). The program aims to reduce pollution released from point sources into navigable US waters, including oceans. Aquaculture facilities that discharge into US waters and produce at least 20,000 pounds of cold water fish or 100,000 pounds of warm water fish per year have been determined to be point sources by the EPA, called Concentrated Aquatic Animal Production (CAAP) facilities [31]. The EPA can also determine if a facility is a CAAP on a case-by-case basis. As CAAP facilities, commercial offshore farms are expected to fall under NPDES and effluent limitation guideline (ELG) requirements [32], but so far the EPA has not required NPDES permits for pilot facilities in the ocean [33]. Allowing pilot facilities to operate in the ocean without NPDES permits requiring best practices, pollution limits, monitoring, and reporting is a missed opportunity to monitor and minimize impacts on the environment and public health [33].

For ELGs to include numeric limits on pollution discharges, the EPA must issue water quality standards for the relevant water body, and they have not been issued for federal ocean waters. Without water quality standards, less stringent ocean discharge criteria (ODC) may form the basis of relevant NPDES permits. ODCs have not been updated since 1980, and although ODCs can be used to require monitoring and to determine if a CAAP facility will cause undue degradation based on the impacts of proposed pollutants, the EPA has not yet defined how they will use ODCs to regulate offshore aquaculture facilities [33]. Limiting and monitoring pollutants released into the ocean is important for public health because drugs and chemicals used in offshore aquaculture can cause food safety issues for consumers of both farmed fish and impacted wild seafood. It is important to note that the NPDES program is largely operated by states that meet EPA requirements, so the EPA must assess and/or build its own capacity in order to regulate aquaculture in the EEZ.

#### 3.2.2. Ocean Dumping Act 

The Ocean Dumping Act (ODA) is Title I of the Marine Protection, Research, and Sanctuaries Act [34]. The EPA has authority over ocean dumping, except disposal of dredged materials, which is regulated by the Department of Defense under the US Army Corps of Engineers. NPDES permits currently incorporate requirements of the ODA [34,35], and it is not clear if there will be separate requirements and/or permits for offshore finfish aquaculture in the EEZ under the ODA. If a separate permit is needed, the requirements will be based on whether the materials released from the site will affect human health, the marine environment, ecological systems, and other economic opportunities [34,35].

#### 3.2.3. Federal Insecticide, Fungicide and Rodenticide Act

The Federal Insecticide, Fungicide, and Rodenticide Act (FIFRA) authorizes the EPA to oversee the sale and labeling of pesticides [36]. Pesticides are used in offshore aquaculture to control insects, crustaceans, worms and plants (e.g., algae) and may require registration with the EPA. For example, copper-based pesticides, which can be used in aquaculture to control algae, are registered through FIFRA [37]. The EPA considers many human health and environmental impacts of a pesticide, including effects on humans, fish, and endangered species. Once registered, pesticides are required to be labeled with approved directions for application, mixing, and storage [36]. These requirements may reduce the risk of health impacts due to occupational exposure and exposure to pesticides through consumption of farmed and wild fish species.

#### 3.2.4. Toxic Substance Control Act

The EPA can issue restrictions and/or require reporting, record keeping, and testing for new and existing chemicals and mixtures in the US under the Toxic Substance Control Act (TSCA) [38]. TSCA does not cover pesticides regulated by FIFRA or chemicals covered by the Federal Food, Drug, and Cosmetic Act. The EPA maintains a large inventory of existing chemicals and relevant restrictions, and can issue rules for new chemicals or new uses for existing chemicals in an effort to reduce exposure and impacts through manufacture, use, and disposal of chemicals [39]. The EPA Office of Inspector General found in 2010 that implementation of TSCA was inhibited by a lack of test data, overreliance on industry to submit data, a lack of resources allocated to implementing TSCA, and a tendency to withhold industry information from the public [40]. Without resolving these issues, TSCA may not play a significant role in regulating chemicals used in offshore aquaculture to protect public health.

### 3.3. Food and Drug Administration (Department of Health and Human Services)

#### Federal Food, Drug, and Cosmetic Act and Relevant Amendments

Oversight granted to the FDA by the Federal Food, Drug, and Cosmetic Act (FFDCA) applicable to aquaculture includes regulation of animal drugs and feed additives. For a new animal drug to be approved, the FDA requires a drug sponsor, typically a pharmaceutical company, to submit information on the drug’s effectiveness, side effects, parameters for safe use, manufacturing process, potential environmental impact, and food safety if the animal is raised for human consumption [41]. Relevant public health considerations include levels of drug residues in food products, occupational exposure during storage and use, health effects that could be caused by environmental contamination, and a potential increase in antimicrobial resistant pathogens on the animal used for food or in the surrounding environment due to the use of certain animal drugs. There are currently 15 drugs approved for use in aquaculture in the US [42], and one food-grade genetically engineered salmon is being evaluated through the drug approval process [43].

The Minor Use and Minor Species Animal Health Act (MUMS) of 2004 amended the FFDCA and created a system of animal drug approvals to encourage the development and sale of drugs that have a smaller market because they treat rare ailments in a major animal species (e.g., horses, cattle, hogs, poultry, dogs, and cats) or treat animals that are classified as a minor species (e.g., farmed fish, ornamental fish, sheep, zoo animals, *etc.*) [44]. Under MUMS, aquaculture drugs can be sold using a conditional approval for up to five years; this approval can be used if the necessary safety data for the drug is complete but effectiveness information is still being compiled. In addition, the FDA considers extra-label use of medicated feeds in minor species a low enforcement priority, even though using medicated feeds for unapproved uses is illegal [44]. The use of conditional approvals and low priority of extra-label use enforcement should be carefully monitored as it relates to aquaculture producers and other farmers since their animals will enter the human food supply. If the US aquaculture industry significantly expands, the minor species designation of farmed fish should be reconsidered.

The Animal Drug User Fee Act (ADUFA) of 2003 was an amendment to the FFDCA to collect fees from animal drug manufacturers to support timely review and approval of new drugs [45]. Following reauthorization of ADUFA in 2008, new amendments to the act directed the FDA to produce annual reports on the quantity of antimicrobials sold for use in US food animal production, including aquaculture, starting in 2009 [45]. For the second reauthorization of ADUFA, there are efforts to request animal drug usage reported by animal class, which would provide more detailed information on drug use in aquatic food animals [46]. Detailed reporting of veterinary drug use in animal production is required in Norway [47], and similar requirements in the US would allow the public health community to track antimicrobial usage and study the impacts.

The Food Quality Protection Act (FQPA) of 1996 was passed to amend FFDCA and FIFRA. Among the changes in FQPA, pesticide residue on any food is now considered unsafe unless the residue falls within an exemption or tolerance. New standards are also set for aggregate yearly and lifetime exposure to pesticides [48]. These standards include stricter requirements for infants and children as determined by EPA, USDA, and DHHS [49].

A rule enacted by the FDA in 1995, drawing on authority granted by the FFDCA, requires the adoption of a Hazard Analysis and Critical Control Points (HACCP) system by seafood processors to improve the safety of consuming seafood [50]. The Fish and Fishery Product HACCP requirement shifts the focus of food safety efforts to prevention by identifying and reducing hazards in critical control points, instead of reacting to outbreaks. Under HACCP, information required from aquaculture producers by processors may include drugs or chemicals used, and testing of water or fish tissue for drug residues, chemical contaminants, or pesticides [50]. The FDA could use this information to study offshore aquaculture and public health risks, or make it available to independent scientists for research.

### 3.4. Occupational Safety and Health Administration (Department of Labor)

#### Occupational Safety and Health Act

The Occupational Safety and Health (OSH) Act established the Occupational Safety and Health Administration (OSHA) as an agency within the US Department of Labor [51]. To reduce workplace injury and deaths, OSHA conducts research and issues requirements regarding exposure limits, use of personal protection equipment, reporting of incidents, training programs, and other requirements that reduce occupational hazards [52]. OSHA has historically regulated aquaculture as an agricultural activity [53]. Importantly, Congress exempts most agriculture operations with fewer than 11 non-family member employees from inspections and enforcement by OSHA [54]. Offshore aquaculture may not fit the agricultural classification due to similarities to commercial fishing and potential involvement of activities such as scuba diving. OSHA does have standards for commercial diving [55]. Additional factors may limit the application of the OSH Act to offshore aquaculture. First, OSHA regulations only apply to certain commercial (*i.e*., fishing, fish processing) and recreational vessels, depending on size; the US Coast Guard generally covers larger vessels [56]. Also, OSH Act jurisdiction effectively ends at the territorial sea because regulations giving jurisdiction over activities in the Outer Continental Shelf to the US Coast Guard and the Bureau of Ocean Energy Management preempt the OSH Act [56]. Therefore, offshore oilrigs and vessels inspected by the US Coast Guard are outside of OSHA’s authority. If OSHA regulates occupational health and safety of offshore aquaculture activities in the EEZ, operations may be exempted due to their classification as an agriculture operation, and for operations not exempted it will represent a new area for an agency that has not focused on occupational issues miles away from the coast.

### 3.5. US Coast Guard (Department of Homeland Security)

#### US Coast Guard (Code of Federal Regulations; Title 46, Chapter 1)

The US Coast Guard is responsible for ensuring the occupational health and safety of individuals working aboard vessels, and the requirements for inspections vary based on what the boat is used for and size/capacity [57]. Certain vessels are required to have specific safety equipment on board, a current logbook, and trained personnel operating the boat [58]. If the US Coast Guard is the lead agency for occupational regulations for offshore aquaculture vessels, issues including exposure to veterinary drugs and chemicals will need to be specifically addressed since the US Coast Guard does not normally oversee agricultural activities.

### 3.6. US Department of Agriculture

#### 3.6.1. Virus-Serum-Toxin Act

The US Department of Agriculture’s (USDA) Animal and Plant Health Inspection Service (APHIS) regulates all veterinary biologics used in the US under authority granted by the Virus-Serum-Toxin Act (VSTA). Veterinary biologics are products derived from living organisms (e.g., bacteria, viruses, spores) and biological processes, and they are used to prevent, diagnose, or treat animal diseases through an immunological process [59]. Examples of veterinary biologics include vaccines, allergens, antibodies, toxins, and diagnostic test kits [60]. APHIS does not regulate antibiotics, steroids, or hormones, which are regulated by the FDA Center for Veterinary Medicine [61]. APHIS is responsible for licensing manufacturing facilities and each product produced, inspection of facilities and records, verification product testing, and permitting imports of biologics. Manufacturers are required to test each batch of product, and keep records of results, to ensure quality and safety [59]. The development of safe and effective finfish vaccines can decrease the use of antimicrobials and reduce public health risks [62]. The VSTA is important for monitoring the quality and safety of veterinary biologics used in offshore aquaculture.

#### 3.6.2. Animal Health Protection Act

The USDA’s APHIS also operates under authority granted by the Animal Health Protection Act (AHPA). The law gives USDA the ability to regulate the import, export, and interstate commerce of all animals that may pose a disease risk to animals produced for food, including farmed fish. AHPA was passed to detect, prevent, control, and eradicate diseases that impact animals produced for food [63]. To achieve these goals, the USDA can hold, seize, treat, or restrict the movement of animals raised on farms [64]. APHIS is also responsible for reporting the occurrence of certain notifiable diseases to the World Organization for Animal Health, and the detection of certain aquatic diseases can impact international trade [64]. A devastating outbreak of infectious salmon anemia and the impact on the Chilean farmed salmon industry in the late 2000’s highlights the critical role of disease detection and control for aquaculture [65]. The AHPA is essential for monitoring diseases among edible farmed fish due to food safety issues and the potential for farmed fish to pass diseases onto wild seafood species. In addition, effective disease control can reduce the use of harmful drugs and chemicals in aquaculture.

### 3.7. Policy With Multi-Agency Jurisdiction

#### National Environmental Policy Act 

The National Environmental Policy Act (NEPA) requires federal agencies to consider the environmental impact of their decision-making in a systematic manner. The lead agency with primary responsibility for carrying out or approving a project must prepare an Environmental Assessment (EA) or Environmental Impact Statement (EIS) to analyze proposed actions that may have an impact on the quality of the environment [66]. An EA is less extensive and can be used to determine if a significant impact is likely. If no significant impact is found, an EIS may not be required [66]. NEPA also requires that each significant impact from the proposed action be identified, along with alternatives to mitigate the effects [66].

The Gulf of Mexico Fishery Management Council included an EIS in the FMP created to manage offshore aquaculture, and the impacts they identified included “increased nutrient loading, habitat degradation, fish escapement, competition with wild stocks, entanglement of endangered or threatened species and migratory birds, spread of pathogens, user conflicts, economic and social impacts on domestic fisheries, and navigational hazards” [12]. The Council included certain requirements in their proposed permitting system to minimize the environmental impacts identified in the EIS. NOAA may conduct their own EIS prior to issuing federal regulations for offshore aquaculture in the EEZ.

## 4. Discussion

Siting and regulating aquaculture facilities involves a complex set of economic, social, and ecological trade-offs, which in some cases includes public health issues [67]. The US federal government has many legislative tools available to regulate offshore finfish aquaculture and minimize public health risks, but the number of laws and agencies potentially involved may serve as barriers to effective regulation. In addition, some agencies may not currently have the capacity or expertise to properly regulate offshore finfish aquaculture. Agencies should be provided the necessary resources and time to develop and coordinate regulations, and the capacity to enforce the regulations, before offshore finfish aquaculture permits are issued. The US government should also consider regulatory approaches of countries with significant near- or offshore finfish aquaculture industries, such as Norway, Canada, Chile, and the United Kingdom. These countries take different approaches to regulating near- and offshore finfish aquaculture [68]. Importantly, researchers studying approaches in various countries have stressed the importance of reporting information about the industry to the public, even if the reporting does not involve regulatory restrictions [69].

Extensive collaboration among multiple agencies is required to oversee the range of regulatory requirements that are needed to protect public health, and some collaboration is already underway. The Joint Subcommittee on Aquaculture (JSA) was formed by the National Aquaculture Act (Table A1) to promote US aquaculture and facilitate inter-agency collaboration. The JSA has no direct regulatory authority, but it helped coordinate an effort by USDA, Department of Commerce (NOAA), and the Department of the Interior (Fish and Wildlife Service) to create the National Aquatic Animal Health Plan for the US [64]. The plan contains useful information and recommendations that can inform aquaculture regulations.

This is the first policy analysis on offshore finfish aquaculture in the US focused on public health. In addition to providing an important perspective on the laws described, some of the laws covered here have not been included in other reports on this topic, which mainly covered environmental issues. This is especially true for occupational health and safety laws and their relevance to offshore aquaculture.

## 5. Conclusions

The federal government is taking steps to develop offshore finfish aquaculture in the US, and it is not yet clear if a large-scale offshore industry can be operated profitably and safely. Federal agencies will need to work collaboratively and transparently in order to ensure that comprehensive regulations are developed that address the risks associated with this type of food production. Our analysis highlights 11 laws that could be used to limit and/or monitor environmental and occupational public health risks related to the use of drugs and chemicals, pollution from waste and uneaten feed, disease transmission, use of feed additives, and occupational hazards in offshore finfish aquaculture. Some of these laws could be overlooked if agencies only consider environmental impacts, therefore, public health professionals should provide input on the development of offshore finfish aquaculture regulations.

## Appendix

**Table A1:** US federal laws relevant to offshore aquaculture with low potential to address public health issues.

Legislation	Agency	Original Purpose	Applicability to Offshore Aquaculture	Applicability to Public Health
1. Coastal Zone Management Act (CZMA)	Dept. of Commerce: NOAA: Office of Ocean and Coastal Resource Management (OCRM)	To encourage better management of coastal resources by states through federally approved coastal management programs [1].	This law has a “Consistency Requirement”, which stipulates that federal agency activities in or adjacent to a state’s coastal zone must be consistent with that states approved coastal management program. A consistency certification may be required with the adjacent state’s coastal management program [1].	This law does not directly impact public health, however, a state has the right to veto a federal agency activity [1] if that activity poses an environmental or public health risk to it’s coastal zone; *i.e.*, is inconsistent with the state’s coastal management plan.
2. Endangered Species Act (ESA)	Dept. of Commerce: NOAA: NMFS administers ESA for marine and anadromous species; Dept of Interior: FWS administers ESA for freshwater species.	To conserve and protect species of animals that are considered endangered (in danger of extinction) or threatened (may become endangered) [2].	Required Consultations with NMFS or FWS (depending on species) regarding the impact of proposed activity on ESA-listed species. Permits or authorizations could be required for aquaculture activities affecting or interacting with ESA-listed species [2].	This law is designed to protect wildlife and is not directly applicable to public health.
3. Fish and Wildlife Coordination Act (FWCA)	Dept. of Interior: FWS;Dept. of Commerce: NOAA: NMFS	This law gives FWS or NOAA the authority to evaluate the impact of any proposed water resource development project on the surrounding wildlife and fish [3].	Consultation with FWS or NMFS or relevant state agency may be required for proposed offshore aquaculture projects [3].	This law is designed to help manage and protect fish and wildlife resources and can be used to regulate environmental impacts that effect wildlife, however, it probably cannot be used to directly address public health risks.
4. Lacey Act	Dept. of Interior: Fish and Wildlife Service (FWS); Dept. of Commerce: NOAA: National Marine Fisheries Service (NMFS) Dept. of Agriculture (USDA): Animal and Plant Inspection Service (APHIS)	To protect wildlife by combatting the illegal transport and trade of wild animals, fish, and plants [4].	An amendment to the Lacey Act in 1981 expanded its application to all wild animals, including animals or fish that were bred or raised in captivity [4]. This law can be used to regulate aquaculture products that are transported or sold in violation of federal, state, or foreign laws [4].	The Lacey Act regulates seafood fraud and mislabeling of seafood products, including aquaculture products [5].
5. Marine Mammal Protection Act (MMPA)	Dept. of Commerce: NOAA: NMFS; Dept. of Interior: FWS	MMPA disallows the take of marine mammals in US waters, as well as the import of marine mammal and related products into the US. Exceptions are granted through authorizations [6].	Authorizations may be required for aquaculture activities interacting with marine mammals [6].	This law is designed to protect wildlife and is not directly applicable to public health.
6. National Aquaculture Act	Dept. of Agriculture (Lead agency); Dept. of Commerce; Dept. of Interior	To promote aquaculture, develop a national aquaculture policy, and develop national aquaculture development plans [7]. Establishment of the Joint Subcommittee on Aquaculture [7].	This law is applicable to all types of aquaculture.	The purpose of this law is to promote aquaculture and facilitate intra-agency cooperation for implementing aquaculture development plans [8]. It is not applicable to the regulation of public health concerns from offshore aquaculture.
7. National Marine Sanctuaries Act (NMSA)	Dept. of Commerce: NOAA	NMSA allows the Secretary of Commerce to designate marine areas of special national importance as national marine sanctuaries [9].	A permit may be required for aquaculture activities interacting with national marine sanctuaries [10].	The purpose of this law is the creation and protection of marine sanctuaries; it is not directly applicable to public health concerns from offshore aquaculture.
8. Outer Continental Shelf Lands Act (OCSLA)	United States Army Corps of Engineers (ACOE); Dept. of Interior: Minerals Management Service (MMS)	The law established jurisdiction over submerged lands in the outer continental shelves [7]. OCSLA also gives the Secretary of the Interior authority for mineral exploration and development in outer continental shelves [11].	This Act extends the RHA by giving ACOE the authority to regulate structures in the EEZ [7]. Under OCSLA, MMS can authorize existing structures or facilities, such as oil platforms, to be used for marine-related activities including aquaculture [11].	This law is not relevant to public health concerns from offshore aquaculture.
9. Rivers and Harbors Act (RHA)	United States Army Corps of Engineers (ACOE); United States Coast Guard (USCG)	The purpose of this law is managing and protecting navigational access in US waters [7].	RHA gives ACOE the authority to regulate structures/devices in federally controlled waters. Additionally, the coast guard has authority to regulate vessel traffic and safety measures such as lighting and signals [7]. Section 10 of the RHA requires permits for structures in navigable waters of the US [12].	This law is not relevant to public health concerns from offshore aquaculture.
